# A decision support system for quality of life in head and neck oncology patients

**DOI:** 10.1186/1758-3284-4-3

**Published:** 2012-02-16

**Authors:** Joaquim J Gonçalves, Álvaro M Rocha

**Affiliations:** 1 GIMED, University Fernando Pessoa, Pr. 9 de Abril 349, 4249-004 Porto, Portugal; 2 EST, IPCA, Campus do IPCA, Lugar do Aldão, 4750-810, Vila Frescainha S Martinho BCL, Portugal

**Keywords:** Heath and Neck Oncologic Patients, Health-Related Quality of Life, Knowledge Management Systems, Decision Support Systems

## Abstract

**Background:**

The assessment of Quality of Life (QoL) is a Medical goal; it is used in clinical research, medical practice, health-related economic studies and in planning health management measures and strategies. The objective of this project is to develop an informational platform to achieve a patient self-assessment with standardized QoL measuring instruments, through friendly software, easy for the user to adapt, which should aid the study of QoL, by promoting the creation of databases and accelerating its statistical treatment and yet generating subsequent useful results in graphical format for the physician analyzes in an appointment immediately after the answers collection.

**Methods:**

First, a software platform was designed and developed in an action-research process with patients, physicians and nurses. The computerized patient self-assessment with standardized QoL measuring instruments was compared with traditional one, to verify if its use did not influence the patient's answers. For that, the Wilcoxon and t-Student tests were applied. After, we adopted and adapted the mathematic Rash model to make possible the use of QoL measure in the routine appointments.

**Results:**

The results show that the computerized patient self-assessment does not influence the patient's answers and can be used as a suitable tool in the routine appointment, because indicates problems which are more difficult to identify in a traditional appointment, improving thus the physician's decisions.

**Conclusions:**

The possibility of representing graphically useful results that physician needs to analyze in the appointment, immediately after the answer collection, in an useful time, makes this QoL assessment platform a diagnosis instrument ready to be used routinely in clinical practice.

## Background

### Scope

The concept of "Quality of Life - QoL" is used in different contexts and situations, reaching practically all sectors of society. The perception that an individual holds about his place in life, which depends upon his culture and values, defines this individual's Quality of Life (QoL). When applied in a health context this is known as: Health-Related Quality of Life (HRQoL) [1]. Nowadays, indicators of HRQoL are used in health management strategies. Managers, economists, political analysts and pharmaceutical companies use QoL measures from the World Health Organization (WHO) in some of their departments [2]. Today, HRQoL is a medical goal, being used in epidemiological studies, clinical essays, medical practice, health-related economic studies, and in planning and comparing measures and strategies [3].

Preliminary studies indicate that the implementation of a patient HRQoL assessment in Portugal is challenged and questioned for several factors involving health institutions, health professionals and patients [4]. The reasons include: a lack of familiarity with relevant studies in this area; the absence of sensitivity; lack of time; reluctance in accepting that the patient's perceptions regarding their own outcomes are as important as the physicians [5]; difficulty in quantifying subjective parameters; difficulty in converting tacit knowledge in explicit knowledge; inexistence of friendly computer-based applications; inexistence of health care service infrastructures that enable a routine HRQoL assessment.

The purpose of this project is to allow the physician to use patient's QoL measurements as clinical decision support elements. A timely knowledge of the patient's QoL-related elements constitutes another factor that may, in certain circumstances, contribute to a better decision making. On the other hand, a systematic patient QoL data collection allows the standardization of this information and to infer therapeutic strategies for a specific patient. By other words, in the presence of several therapeutic strategies this can help the physician by giving him clues about the patient's future QoL according to applied medical acts.

In the this paper we intend to demonstrate the importance of HRQoL assessment in oncologic patients, and the relevance of Knowledge Management Systems (KMS) as decision making aids. We analyze this problem and show the results obtained with a platform developed for the self-evaluation questionnaire that measures patients QoL and collects clinical information in order to infer about the patient future Qol through crossing the QoL measure and the several treatments used in the patients.

### Evaluation of HRQoL in oncologic patients

Malignant tumors are the second leading cause of death in Portugal. Their relevance as a morbidity and mortality factor is growing and their social impact is being recognized [1]. The global weight of oncologic disease is growing, given the economic and social costs involved in the prevention, treatment and rehabilitation of it [6].

Research methods used in oncology enable us to analyze the oncologic process in its physiopathologic and clinical aspects, penetrating wide domains such as psychological, social, economic and organizational domains [1]. Epidemiology and statistics are significant areas of this study, since oncologic care can only be programmed through safe databases [7]. Assessing the implementation of these diseases in our community helps to recognize the global impact of tumors and to evaluate the effectiveness of the adopted control measures [2].

The time where therapeutic decisions were not discussed with the patient and the family, and treatment options were not even considered, has long since passed. Oncologic patients were frequently not informed of their diagnosis after their families were. This reality has changed and, today, patients participate, or should participate, in the several stages of their treatment [1].

In fact, patients motivated to participate in their treatment and rehabilitation plan often show a better QoL, and should therefore be involved in the strategies developed to fight their disease. Furthermore, evidence shows that a global patient QoL optimization can lead to a higher survival rate and to a higher quality of life [1].

Promoting the integration of QoL assessment in clinical practice can result in the optimization of infrastructures and methods capable of improving patients QoL [8]. A validated, safe and scientifically-based measuring instrument must be made available in a simple format, understood both by the patient and the physician, for being completed in less than 10 minutes [9].

Although being a subjective concept, HRQoL is quantified objectively and does not merely represent the inexistence of disease [10]. The multidimensional conception of HRQoL comprises a wide range of physical, psychological, functional, emotional and social variables, which as a whole, define welfare [11]. These domains vary individually according to religion and beliefs, culture, expectations, perceptions, education, knowledge, etc. [11].

Table 1 represents schematically the mains HRQoL dimensions and items, proposed by the WHO [12]:

**Table 1:** Dimensions and items for HRQoL assessment

QoL Domains			
**Physical Health**	**Psychological**	**Social Relationships**	**Relationship with the Environment**

Activities	Self-Esteem	Sexual Activity	Economy
Pain	Spirituality	Social Support	Information
Dyspnea	Body Image	Family	Means of
Mobility	Thoughts	Personal	transportation
Medication	Negative Feelings	relationships	Security
Insomnia	Positive Feelings		Services
			Free time

### KMS in routine HRQoL assessment

Preliminary studies on oncologic patients conclude that the use of an adequate software for the HRQoL assessment, data collection and processing, allows us to obtain self-answered questionnaires from patients, an automatic quotation of these questionnaires, the creation of a database and the statistic analysis of the results, performing a routine HRQoL clinical assessment [13].

Moreover, the graphical representation of results enables a fast patient HRQoL assessment by the physician, and this evaluation becomes a diagnosis instrument to be used in routine clinical practice [13].

HRQoL assessment is dynamic and requires periodic reevaluations [14]. It should be done objectively and quantitatively on a routine basis. Then, the selection of a measuring instrument with good psychometric characteristics, easy to administrate and to quantify, that doesn't increase the appointment time and with a multidimensional character is most important. It must be answered and quoted before appointments. The results should remain confidential and anonymous, and when graphically represented should allow an easy reading of the patient's self-perception. Thus, HRQoL assessment becomes a diagnosis instrument that identifies patient's problems, highlights certain signs and symptoms that could otherwise go unnoticed, improves the physician-patient communication and assists therapeutic decisions; in other words, it renders the appointments easier. By analogy, the physician can evaluate the evolution of his patient's state comparing two or more assessments obtained in different periods [15].

However, a routine assessment implies the design of a new appointment protocol. The analysis and specification of the information system requirements, as well as the specification of necessary activities for the process, define the knowledge management system which supports the clinical decision aid system, based on the HRQoL assessment.

### Methodology

A software platform to study the quality of life of oncology patients was designed and developed in an action-research process with patients, physicians and nurses.

In order to assess the impact created by the application in the given answers we randomly selected patients from the otorhinolaryngology service in Oporto's IPO (Portuguese Institute of Oncology). We selected fifteen days from May, June and July of 2011, and all patients attending consultations on those days were invited to participate in this study. All of them accepted the invitation and then we obtained a sample of 54 individuals (Table 2). These patients answered the same questionnaire twice, one in paper form - the traditional model - and the other on the computer using the software developed for that purpose, with 40 minutes temporal gap. Half of the patients answered first on the paper form and the other half answered first on the computer platform, the minimum time between answers was 40 minutes. In both cases the answer time was measured and the patient's preference between the paper and the computer was registered. Information regarding patient's affinity with computer use was also registered.

**Table 2:** Patients demographics

Gender	Age	
Male (37) 68,5%	Mean	57,0
	
	95% Confidence Interval for Mean	[52,9;61,1]
	
	Std. Deviation	12,3
	
	Minimum	22
	
	Maximum	88

Female (17) 31,5%	Mean	63,7
	
	95% Confidence Interval for Mean	[56,6;71,0]
	
	Std. Deviation	14,0
	
	Minimum	40
	
	Maximum	79

In order to understand if the computer-based environment influenced or not the answers we analyzed the obtained values for each given answer, in both of the assessment moments, using a collection of statistical models and tests. Answers obtained in paper support and through the computer-based platform were matched. To understand if the computer-based platform did not influence the patients answers we hypothesized that distributions, for each variable in study, were identical. We first tested the entire set of answers and then two subsets, which divided patients that answered firstly on paper and patients that answered firstly on the computer.

In the validation process two standardized questionnaires were used, both from EORTC (European Organisation for Research and Treatment of Cancer): QLQ-C30 and QLQ-H&N35. The first one is a global questionnaire developed for all types of oncologic patients. It has thirty questions grouped in five domains (physical, social, emotional, functional and cognitive). The second is a specific questionnaire for Head and Neck oncology patients, with thirty five questions.

The two statistical hypotheses for a bilateral test in each situation were written.

$$\text{Hypothesis}\;{\text{H}}_{0}\;\text{F}\left({\text{X}}_{0}\right)=\text{F}\left({\text{X}}_{1}\right);\;\text{Hypothesis}\;{\text{H}}_{1}:\;\text{F}\left({\text{X}}_{0}\right)<>\text{F}\left({\text{X}}_{1}\right).$$

We used the Wilcoxon test, the most appropriate when the dependent variable is measured in an ordinal scale [16]. In both of the questionnaires (QLQ-C30 and QLQ-H&N35) adopted to evaluate the QoL the test results did not allow to conclude if there were significant differences between distributions, for the two samples and the three mentioned situations.

A high level of significance was always attained, independently of the global or the partial analysis of the sample, divided between those who firstly answered on paper and those who firstly answered on the computer, so the hypothesis of not existing a significant difference between answers was accepted. We can thus state that the software use does not influence patient's answers.

After the validation of the platform to obtain a patient self-assessment with standardized QoL measuring instruments, we adopted and adapted the mathematic Rash model to make possible the use of QoL measure in the routine appointments.

The Rasch model estimates the question difficulty level and the person ability level with an iterative process. This process takes a lot of time and it is not compatible with a routine appointment. Thus, it was necessary to understand how we could make the process faster while maintaining accuracy in the values obtained for the estimated parameters.

We analyze the running time by varying the number of iterations and the sample size without losing accuracy. So, it was possible to determinate the sample size and the number of iterations to calculate the parameters that minimize the execution time.

In addition, we determine what were the calculations that could be made in advance, that is, the calculations that could be made before the appointment. Thus, the time required for QoL assessment was reduced to five minutes and you can use it in a routine assessment.

## Results and discussion

### Friendly software design

It cannot be denied that the health domain is extremely sensitive, and every aspect that interferes with traditional processes is potentiated in terms of impact. Thus, we started this project assessing the influence that a technological environment would have in the patient's behavior.

Knowledge management systems can and should be used in order to optimize certain procedures, but the type of organization where they are introduced in must be kept in mind. Dimensions and items for a model of knowledge management were presented in Table 1.

The purpose of this project involves the development of an informational platform that would not interfere with patient's answers when used, and which could be applied by health professionals. This software should run through a browser working in the health unit's intranet, or even in the internet.

The main requirement in the creation of this software was to build an interface as close to a traditional paper form as possible. Using key-words like usability, accessibility and confidentiality, the intention was to build a simple interface with an intuitive use, where the correction to an answer could be done in a clear, objective way, where the patient could clearly understand the confidentiality of his answers, and accessible to all types of patients. Blind, illiterate and physically challenged individuals are recurrent amongst the frequent oncology service patients of the IPO in Oporto. Sound or touch screens are presently the two used interface solutions, but we are still investigating the use of other communication devices.

**Figure 1:**
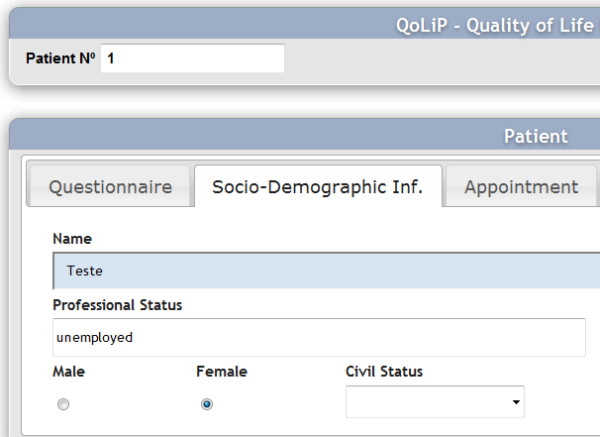
**View of the QoL screen**.

**Figure 2:**
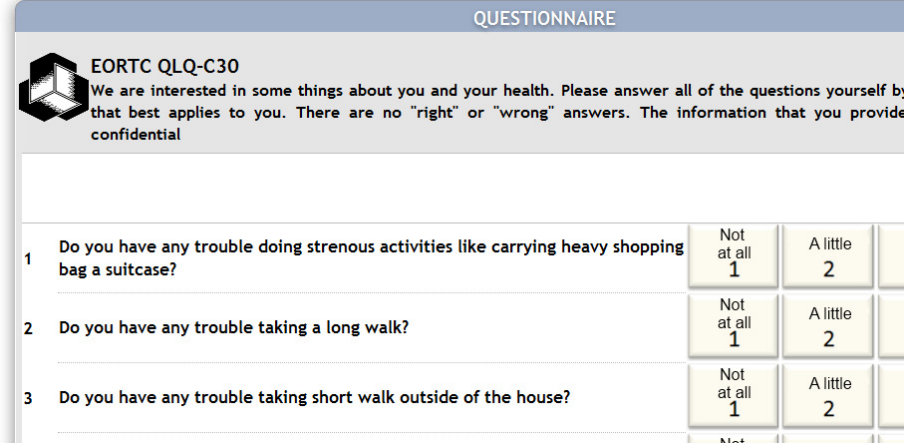
**View of the patient when answering the questionnaire**.

Next, we present two figures. Figure 1 shows a view of the QoL screen and Figure 2 shows a view of the patient's screen when he answers the questionnaire.

### Computer versus paper support

**Figure 3:**
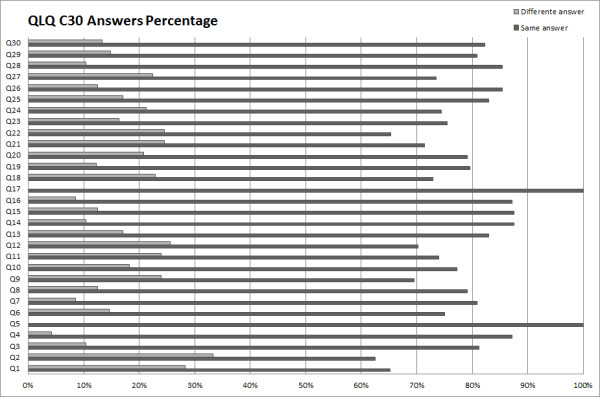
**QLQ-C30 Answers Comparison**.

**Figure 4:**
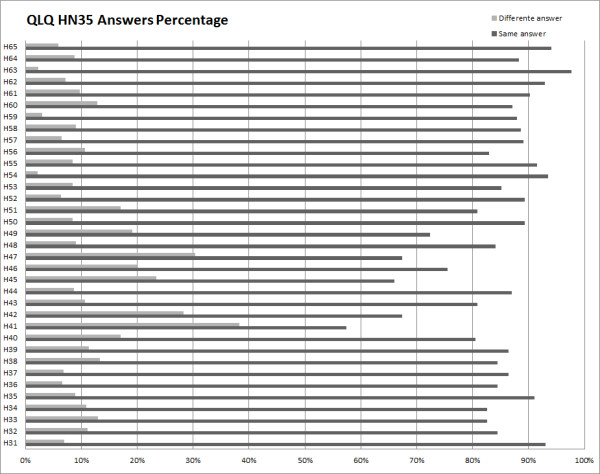
**QLQ-HN35 Answers Comparison**.

The following graphics (Figure 3 and 4) show, for each question, the percentage of equal and different answers given by patients answering on the computer, comparing with answers given on paper.

We thus concluded that the answers given by patients on paper and on the computer-based platform are generally the same. Answers q1, q2, q22 of QLQ-C30 and answer h41 of QLQ-H&N35 show the higher number of different answers, a little over 40% in the case of h41 and fewer than 40% on the other cases.

It is worth noting that the specific questionnaire (QLQ-H&N35) reveals a higher proportion of equal answers. Ideally, the answers should always be the same, but previous experiments (performed on paper) show that answers given by patients in two separate moments are sometimes different, and the percentage for this difference is close to the one we observed between the answers on paper and the answers on the computer. This confirms the results observed in the mentioned test, leading us to conclude that the different results are caused by other factors.

The Wilcoxon test results (Table 3 and Table 4) suggest the not rejection of null hypothesis, in fact for all answers the significance level is minor that 0,05. So we cannot conclude that the answers are not similar between paper support and computer support.

**Table 3:** Wilcoxon test results for QLQ-C30

	q1	q2	q3	q4	q5	q6	q7	q8	q9	q10
Asymp. Sig. (2-tailed)	0,87	0,23	0,51	0,08	1,00	0,16	0,22	0,08	0,23	0,33
	
	q11	q12	q13	q14	q15	q16	q17	q18	q19	q20
	
	0,19	1,00	0,48	0,74	1,00	0,08	1,00	0,79	0,71	0,53
	
	q21	q22	q23	q24	q25	q26	q27	q28	q29	q30
	
	0,17	0,18	0,39	0,61	0,56	0,53	0,26	0,39	0,79	0,17

**Table 4:** Wilcoxon test results for QLQ-H&N 35

	h31	h32	h33	h34	h35	h36	h37	h38	h39	h40
Asymp. Sig. (2-tailed)	0,56	0,79	0,66	0,94	1,00	0,44	0,92	0,06	0,32	0,76
	
	h41	h42	h43	h44	h45	h46	h47	h48	h49	h50
	
	0,44	0,54	0,58	0,08	0,91	0,48	1,00	0,30	0,51	0,25
	
	h51	h52	h53	h54	h55	h56	h57	h58	h59	h60
	
	0,25	0,89	0,55	0,59	0,32	0,47	0,68	1,00	0,36	1,00
	
	h61	h62	h63	h64	h65					
	
	0,32	0,56	0,32	0,71	0,16					

The rejection of null hypothesis do not guarantee that answers are similar, so we analyze the correlation between both answers, and we create a linear regression line (y = mx + b) to analyze the coefficient of dependent (m) variable and the independent term (b).

The linear regression analysis allowed conclude for all questions that the coefficient of dependent variable value was near from one (1) and the independent term value was near from zero (0). The worst obtained value was in question 16 where the values were 1,038 and 0,017 respectively. The correlation coefficient was 0,853. Therefore we realized a *t-Student *test to verify if the coefficient of dependent variable value could be zero in the population. The test allowed to reject the null hypothesis. In resume, can be concluded that the answers are similar for both supports, i.e., the platform does not influence the patient's answers.

**Figure 5:**
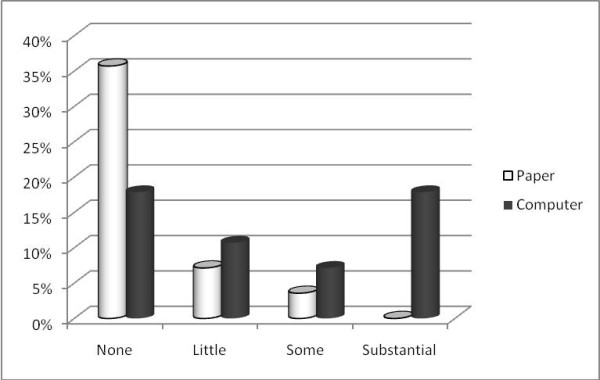
**Patient's preferences by patient utilization level**.

Additionally was asked to the patients data about their impression with the platform in comparison with the paper. So, we asked about their level like user in four categories (none contact, a little contact, some contact and substantial contact) and what they prefer: the paper or the computer. The results are showed in Figure 5.

It is clear that except the patients that never use the computer, the preference goes to the answer in the computer platform. It should be emphasized the relationship between the level of computer use and preference for answering in this way.

### Data analysis

After the data registration stage, concerning the patient's QoL, it is important to forward this information in a clear and objective way to the physician, to enable an improved decision making. The following stage was the clinical variables identification and development of the output information obtained from physicians contributions.

Measures verified in clinical analysis differentiate patients from each other, but we understand that QoL measuring should also be considered in patient standardization.

We used the Rasch model [17] to analyze the patient answers. An important feature of the Rasch model is the sufficient raw score consistent estimation of item parameters without reference to the distribution of the latent variable in the possible population. This feature allows to analyze each answer from each patient individually without concern with the other answers or the population distribution.

**Figure 6:**
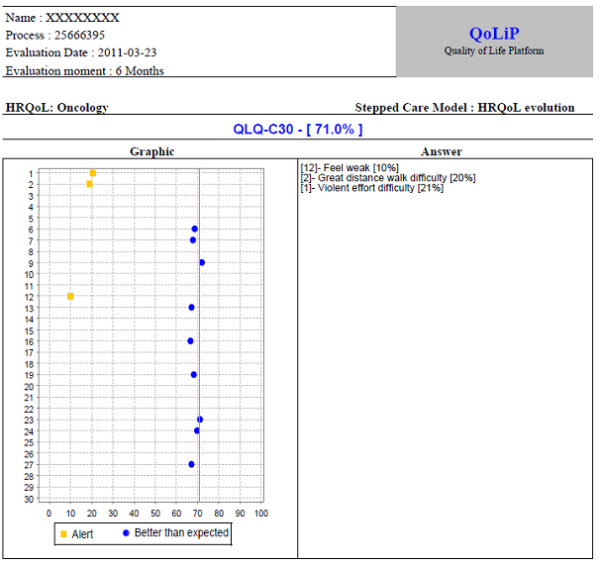
**Graphic with patient's answers**.

Figure 6 shows a graphic with patient's answers: signaled in yellow are the answers below what is expected for this type of patient, and signaled in blue are those above the expected. In the right column are highlighted the most critical answers in order to turn easier and quick the physician understanding.

The advantage in using of this platform is almost the quick analysis by the physician about patient clinical problems. In fact, the physician takes note about patient problems before observing the patient. This information facilitates and improves the conduction of the appointment. Without using the platform, it is not possible to identify some signs and symptoms.

### Clinical decision support system

Why do we consider this system as a clinical decision support system?

In addition to the information about QoL the platform can register the patient's clinical information and socio-demographic characteristics allowing to classify and group patients according to these characteristics.

The collected data can help the physician in two levels:

• Identify the problems that the patient has at the moment;

• Assist the physician in decision-making by providing a forecast on future quality of life of the patient according to the treatments prescribed.

It is important that the patient and the physician know the effect that a treatment will have within 3, 4 or 5 years in his QoL. The decision about choosing a treatment protocol should include the patient's QoL not necessarily during the treatment but especially during the years following it. The objective measurement of patient's QoL allows, in this context, to consider it as a clinical data contribution to the characterization of the patient.

## Conclusions

In this paper we defined the concept of QoL, in different contexts and situations, which is reaching almost every sector of society. The main focus, however, was on the Health context.

Some studies have suggested that the implementation of a patient HRQoL assessment in Portugal is challenged and questioned for several factors involving health institutions, health professionals and patients [4].

The platform designed and developed in this project gives the physician an opportunity to use patient's QoL measurement in real time as a clinical decision support element.

The knowledge about patient QoL constitutes another factor that may, in certain circumstances, contribute to a better decision. The systematic patient QoL data collection allows the standardization of this information and to infer therapeutic strategies for a specific patient. Moreover, therapeutic alternatives can help the physician by giving him important data from which he can infer the patient's future QoL.

We proved the validity of the developed platform in the acquisition of data required for the QoL assessment, and in allowing a routine QoL assessment to become a part of the appointment.

An evolution of the platform for collecting clinical information, in order to typify patients and therapies according to a specific patient's QoL and a class of patients, is under development. The need to develop this platform underlines the importance of knowledge management systems as decision making aids.

## Competing interests

The authors declare that they have no competing interests.

## Authors' contributions

JG conceived and developed the decision support system, participated in the acquisition, analysis and interpretation of data, performed the statistical analysis, and drafted the manuscript. ÁR participated in the design and coordination of the project, helped to draft the manuscript, and revised it critically for important intellectual content. All authors read and approved the final manuscript.
